# Amorphous Silicon Carbide Platform for Next Generation Penetrating Neural Interface Designs

**DOI:** 10.3390/mi9100480

**Published:** 2018-09-20

**Authors:** Felix Deku, Christopher L. Frewin, Allison Stiller, Yarden Cohen, Saher Aqeel, Alexandra Joshi-Imre, Bryan Black, Timothy J. Gardner, Joseph J. Pancrazio, Stuart F. Cogan

**Affiliations:** 1 Department of Bioengineering, University of Texas at Dallas, Richardson, TX 75080, USA; christopher.frewin@utdallas.edu (C.L.F.); axs169031@utdallas.edu (A.S.); saher.aqeel@utdallas.edu (S.A.); alexandra.joshi-imre@utdallas.edu (A.J.-I.); bjb140530@utdallas.edu (B.B.); joseph.pancrazio@utdallas.edu (J.J.P.); stuart.cogan@utdallas.edu (S.F.C.); 2 Department of Biology and Biomedical Engineering, Boston University, Boston, MA 02215, USA; yardenc@bu.edu (Y.C.); timothyg@bu.edu (T.J.G.)

**Keywords:** amorphous silicon carbide, neural stimulation and recording, insertion force, microelectrodes, neural interfaces

## Abstract

Microelectrode arrays that consistently and reliably record and stimulate neural activity under conditions of chronic implantation have so far eluded the neural interface community due to failures attributed to both biotic and abiotic mechanisms. Arrays with transverse dimensions of 10 µm or below are thought to minimize the inflammatory response; however, the reduction of implant thickness also decreases buckling thresholds for materials with low Young’s modulus. While these issues have been overcome using stiffer, thicker materials as transport shuttles during implantation, the acute damage from the use of shuttles may generate many other biotic complications. Amorphous silicon carbide (a-SiC) provides excellent electrical insulation and a large Young’s modulus, allowing the fabrication of ultrasmall arrays with increased resistance to buckling. Prototype a-SiC intracortical implants were fabricated containing 8 - 16 single shanks which had critical thicknesses of either 4 µm or 6 µm. The 6 µm thick a-SiC shanks could penetrate rat cortex without an insertion aid. Single unit recordings from SIROF-coated arrays implanted without any structural support are presented. This work demonstrates that a-SiC can provide an excellent mechanical platform for devices that penetrate cortical tissue while maintaining a critical thickness less than 10 µm.

## 1. Introduction

Penetrating microelectrode arrays (MEAs) that stimulate or record neural activity usually consist of a base substrate material which may be an insulator or conductor. Typical conducting substrates include silicon [1], tungsten, iridium wire [2,3], and carbon fiber [4,5,6,7], which provide the backbone and structural stiffness necessary to penetrate neural tissue. For the Utah array, silicon is doped to provide conductivity [8], and is usually insulated so that current conduction is restricted to the doped silicon. A common polymeric coating used to isolate the conducting substrate from the surrounding electrolyte is Parylene C. It is also common practice to use thin-film dielectric materials, such as low pressure chemical vapor deposited (LPCVD) SiO_2,_ to encapsulate polycrystalline silicon traces [9]. In most cases another dielectric material, such as Si_3_N_4,_ is deposited over the SiO_2_ to control the intrinsic compressive stress in the SiO_2_ [10,11] or to create a multilayer passivation stack of PECVD SiO_2_/Si_3_N_4_/SiO_2_ over the conducting trace [12]. The silicon - based microelectrodes, however, have been shown to deteriorate when chronically implanted [13,14,15]. Failure modes associated with silicon - based MEA degradation were recently described following array implantation in non-human primates [14].

Recent studies have shown that flexible neural interfaces may provide an alternative to traditional silicon-based implants and have the potential to greatly improve the chronic longevity of the implanted microelectrodes [2,16]. Polymers such as polyimide [17,18], Parylene-C [19,20], SU-8 [21], polydimethylsiloxane (PDMS) [22], and shape memory polymers [23,24] have been investigated as substrates for neural stimulation and recording microelectrodes. Their low Young’s modulus reduces the mechanical mismatch between neural tissue and the implanted device. Thin-film metal conducting traces such as gold or platinum are used between layers of the polymer substrate connecting electrode sites and bond pads. The insulating layers effectively sandwich the conducting traces. Electrode sites are then created by removing or etching the top layer through a precise and controlled microfabrication process. 

Implantation of some penetrating polymer-based MEAs have been aided by a delivery vehicle [5,25,26,27,28] or temporary support structure [5,29,30,31] to minimize buckling during insertion by increasing the critical buckling load [32]. To penetrate neural tissue without the assistance of support structures, a minimum cross-sectional dimension of the shank (the part that penetrates the neural tissue) is typically greater than 20 µm [18,21,33]. Unfortunately, this cross-sectional dimension may still be higher than that required to ameliorate the foreign body reaction, noting that the prevailing thought has been that the minimum geometric dimension requirement, at least in one dimension, should be under 10 µm [34]. We recently described the development of multielectrode arrays based on PECVD amorphous silicon carbide (a-SiC) [35]. Amorphous SiC was chosen because it exhibits robust chemical inertness [36], high electronic and ionic resistivity [37], biocompatibility [37,38,39,40], and is amenable to thin-film fabrication processes [35]. Crystalline SiC has also been used as a material in the fabrication of MEAs and, because it is a wide bandgap semiconductor that can be doped for electronic conductivity, it may be used for conductive traces or as a low-impedance electrode, as well as an insulator [41,42,43,44,45]. The 16 channel MEAs were developed with two a-SiC layers sandwiching a thin-film Au conducting trace. Each shank was 10 µm wide and 2 mm long and had a shank cross-sectional area below 45 µm^2^. The greatly reduced shank cross-sectional dimensions may promote compliance with neural tissue when implanted [46]. The electrode sites were opened at the distal tips by removing the top a-SiC layer and were coated with sputtered iridium oxide films (SIROF) or titanium nitride (TiN) to reduce electrode impedance [35]. 

Here, we evaluate different approaches of reducing the critical buckling load of a-SiC MEAs having individual shank cross-sectional area below 45 µm^2^, and demonstrate insertion of multiple a-SiC MEA shanks into rat cortex. Acute extracellular neural recording from the a-SiC MEAs following array insertion is also presented.

## 2. Materials and Methods 

### 2.1. Thin Film Deposition and Array Fabrication

**Figure 1:**
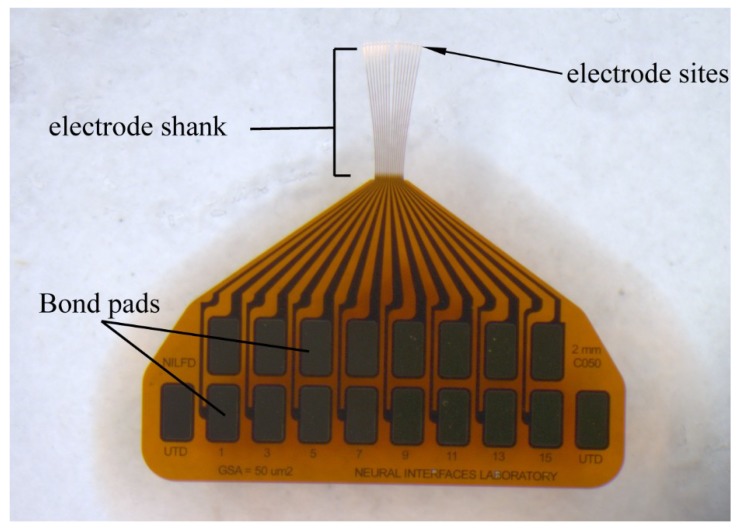
Example of the 16-channel a-SiC microelectrode array (MEA) showing bond pads at the proximal end, 2 mm long electrode shanks, and electrode sites located at the distal tips.

**Figure 2:**
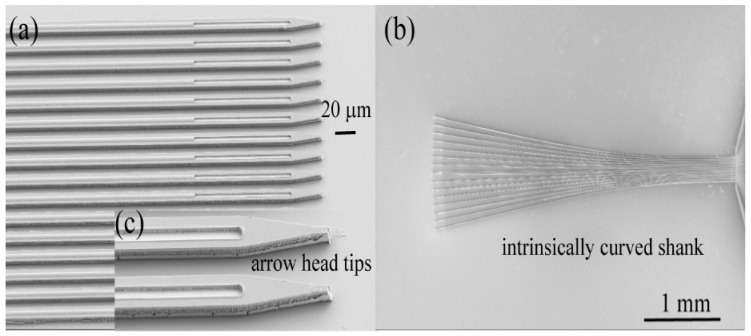
Scanning electron micrographs of as-fabricated 16 channel a-SiC intracortical ultramicroelectrode arrays with straight shanks of identical length (**a**) or intrinsically curve shanks (**b**). Tip profile and electrode site opening are shown in (**c**).

Plasma enhanced chemical vapor deposited a-SiC films using the Plasmatherm Unaxis 790 series deposition system are used as substrates for MEA development. The a-SiC films are deposited at 1000 mTorr, 350 °C, and 0.27 W/cm^2^ using a SiH_4_:CH_4_ gas ratio of 1:3. A 2 µm or 4 µm thick a-SiC film forms the bottom layer of the MEA. The bottom a-SiC layer is followed by the deposition of approximately 350 nm thick patterned gold layer that forms the interconnecting traces. A thin (<50 nm) film of titanium is deposited as an adhesion layer between the a-SiC and gold on both surfaces of the metal to form a trilayer metal structure of Ti/Au/Ti. A second 2 µm a-SiC layer was deposited over the metal traces and bottom a-SiC layer to produce either a 4 µm or 6 µm thick a-SiC superstructure. The details of the fabrication have been reported previously [35]. Briefly, a 1 µm polyimide (HD Microsystems PI 2610) release layer is spin-coated on to a 100 mm silicon wafer and cured at 350 °C under N_2_ for 1 h. The bottom a-SiC layer is deposited on the polyimide followed by a bilayer photolithography process, using LOR5A (Microchem Inc., Westborough, MA, USA) and Shipley S1813 (Microposit, Marlborough, MA, USA) photoresists, to define the metallization pattern. The metal was sputtered or evaporated, and the sample soaked in EBR-PG (Microchem Inc. Westborough, MA, USA) to complete the lift-off process. The second a-SiC layer was then deposited over the metallization and the bottom a-SiC the complete the thin-film stack. The 350 °C deposition temperature of the second a-SiC results in an increase in tensile stress of the metallization by about 400 MPa, for either the evaporated or sputtered trilayers. For the overall device, the effect of the increase in metal tensile stress is a reduction in the overall device stress from about 100 MPa compressive to near-neutral (<20 MPa compressive), recognizing that the overall stress in the device is dependent on the thickness and processing of the individual layers. Another photolithography process, using a positive photoresist, was used to define the electrode sites, bond pads and shape of the individual devices on the wafer. The devices were then formed by reactive ion etching of the exposed a-SiC in SF_6_ plasma using an inductively coupled plasma (ICP) etcher. After the etching process, the remaining resist was stripped and the wafer with the a-SiC MEAs are soaked in deionized water until the arrays release. An example of a 16-channel MEA fabricated by the process described is shown in Figure 1. The device is intended for intracortical studies with only the 2-mm long distal shanks penetrating the cortex. Photolithographic patterning provides a means of creating a variety of array geometries including straight and curved shanks (Figure 2). 

### 2.2. Buckling and Insertion Mechanics

Force measurements were made using a 20 g S-Beam load cell (Futek Advanced Sensor Technology, Inc., Irvine, CA, USA) mounted to a pneumatically controlled micro-positioner (Model 2650 Micropositioner, Kopf Instruments, Tujunga, CA, USA) which has predefined speed settings ranging from 1 µ/s to 4 mm/s. The micromanipulator is hydraulically driven and thus the motion is continuous. The steps in the forcetime curves are due to the sampling frequency of the recording equipment used to measure the load cell output. The sample probe was mounted on a screw which was directly threaded into the bottom of the load cell so that compression forces could be measured as the MEA was inserted into the brain tissue. Before implantation, the probe was lowered until it was directly above the surface of the brain. The load cell was then tared, and the probe inserted 2 mm into the brain at a constant rate of 50 μm/s. For the measurements of buckling forces on glass substrates, the load cell was tared with a slight compressive stress on the tip and then retracted from the surface. This procedure results in an initial tensile deflection in the force-time curve immediately prior to the probe tip striking the glass surface.

### 2.3. Surgery and a-SiC Implantation

All surgical procedures were performed under the approval of the University of Texas at Dallas Institutional Animal Care and Use Committee (IACUC). Long Evans rats were deeply anaesthetized with 5% isoflurane vapor and administered an intraperitoneal KXA cohort consisting of ketamine (65 mg/kg), xylazine (13.33 mg/kg), and acepromazine (1.5 mg/kg) cocktail. The anesthesia was maintained at 0.5 to 1.5% throughout the remainder of the procedure. A 1 to 2 mm square craniotomy was centered 2.5 mm rostral and 2.5 mm lateral to bregma, and bone debris was carefully removed using sterile phosphate buffered solution (PBS). The dura was reflected using a dura pick and the surface of the brain was kept moist with sterile PBS. The Omnetics 18 pin male connector attached to the a-SiC cortical implant was placed within a NeuroNexus IST implantation tool (NeuroNexus, Ann Arbor, MI, USA) and loaded onto a Kopf Model 2650 hydraulic micropositioner (David Kopf Instruments, Tujunga, CA, USA). The implant was inserted to a depth of 1.5 to 2 mm from the cortical surface at an insertion rate of 50 µm/s at a location at the center of the craniotomy, deviating only enough to avoid large surface vasculature. The dura was sealed using Kwik Cast silicone elastomer (World Precision Instruments, Saratosa, FL, USA), followed by a layer of GLUture Octyl/Butyl cyanoacrylate glue (World Precision Instruments, Sarasota, FL, USA). A protective head cap was constructed using two-part dental cement (Stoelting Co., Wood Dale IL, USA) which served to secure and support the implant as well as protect the surgical site. The scalp wound was sutured, and the animal was administered an intramuscular injection of Cefazolin (5 mg/Kg), a subcutaneous injection of sustained release Buprenorphine (0.15 mg/Kg), and 2 mL of 0.9% saline. The rat was individually housed following implantation. Clavamox was administered orally and buprenorphine was administered every 72 h for one week.

### 2.4. In Vivo Recording and Analysis

Following construction and curing of the surgical head cap, recordings for a period of 10 min were collected using an OmniPlex Neural Acquisition System (Plexon Inc., Dallas, TX, USA) connected to the a-SiC array via Omnetics connector and a 16-channel digital headstage. Wideband signals (0.1–7000 Hz) were recorded simultaneously from all 16 electrodes at 40 kHz sampling frequency and later filtered offline using a 4-pole Butterworth high pass filter (250 Hz). A −4σ threshold based on RMS noise calculations was applied to filtered continuous data to identify potential waveforms (or spikes). Single units were identified manually based on 2D principal component clustering using Plexon’s Offline Sorter software (Plexon, Dallas, TX, USA). Sorted units which were not comprised of at least 100 individual spikes or which exhibited greater than 0.5% spike refractory period violations were excluded from analysis. Signal-to-noise ratios (SNR) were calculated by dividing the mean peak-to-peak amplitude of each unit by the adjusted RMS noise of the associated channel, which excluded values greater or less than ±4σ of the filtered continuous signal. 

## 3. Results and Discussions

The 16-channel a-SiC MEAs were generally designed to mate with the 16-channel Omnetics connectors (A79040-001, Omnetics, Minneapolis, MN, USA). Gold bonding pads located at the proximal end of the MEA superstructure, 750 × 500 µm dimensions and pitch of 635 µm ensured that the 16 a-SiC channels mated well with the connector. A solder reflow process using an indium-tin eutectic solder paste consisting of 52% In to 48% Sn (IND.1E, Indium Corporation, Clinton, NY, USA) was used to bond the pads on the connector to the gold bond pads on the MEA. 

To characterize the functionality of the a-SiC platform, MEAs consisting of 16 penetrating shanks with one electrode per shank were fabricated (Figure 1). Each shank was 4 or 6 µm thick, 2–4 mm long, and 7–10 µm wide with 25 µm intershank separation. The shanks were designed with a straight outline and with ‘arrow head’ tip geometry. The shanks are sometimes intrinsically curved with the expectation that such geometry will direct the deployment of the shanks to a larger volume of brain tissue when implanted.

Figure 2 shows shank arrangements of the as-fabricated 16 channel a-SiC penetrating MEAs with (a) straight shanks of identical length and (b) intrinsically curve shanks. Tip profiles are shown in (c). Metal traces are 2 µm wide and run centrally along the length of the shank. The electrode sites are located at the distal tip and are constrained in size and shape by the width of the shanks, such that the 50 µm^2^ electrode sites were 2 µm wide and 25 µm long. 

### 3.1. Insertion of Ultrathin Shanks into Cortex

#### 3.1.1. PEG-Stabilized Shanks

While shanks with very small cross-sectional area offer the promise of reduced FBR, insertion of individual shanks into the neural tissue is challenging. Coating the shanks with polyethylene glycol (PEG) that temporarily stiffens the shanks while leaving a small portion of the tips exposed [5] is an approach previously shown to successfully aid insertion. The PEG coating increases the buckling threshold of the shanks and allows the arrays to be implanted. Using this method, we have inserted 4 µm thick versions of the a-SiC arrays into rat brain. 

**Figure 3:**
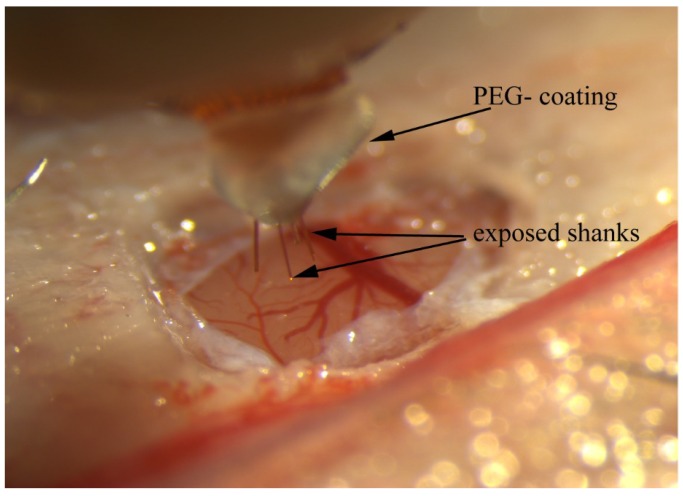
Insertion of a PEG-stabilized a-SiC MEA into rat motor cortex. The PEG temporarily provides mechanical support to the 4 µm thick a-SiC shanks prior to insertion. An insertion rate of 50 µm/s ensures that the PEG completely dissolves as the array is advanced into the brain.

An example of an array coated with PEG (MW 2000, Alfa Aesar, Tewksbury, MA, USA) prior to implantation is shown in Figure 3. Prior to PEG coating, the assembled a-SC array is placed on a mineral oiled aluminum surface. A single flake of PEG is placed on the proximal end of the separated shanks. The PEG is then melted onto the shanks with a soldering gun. As shown in Figure 3, the PEG coating was only used to strengthen the shanks towards the base of the MEA leaving the tips free to individually penetrate the brain. An insertion rate of 50 µm/s was used to insert the shanks so that, as the array is slowly advanced into the brain, the PEG coating dissolves on the surface of the brain without itself penetrating the tissue, preserving the sub 10-µm dimensions of the shanks that are inserted into the brain. Based on visual observation with a surgical microscope, the shanks appear to penetrate the parenchyma of the brain without dimpling the cortex.

#### 3.1.2. Bundled Shanks

**Figure 4:**
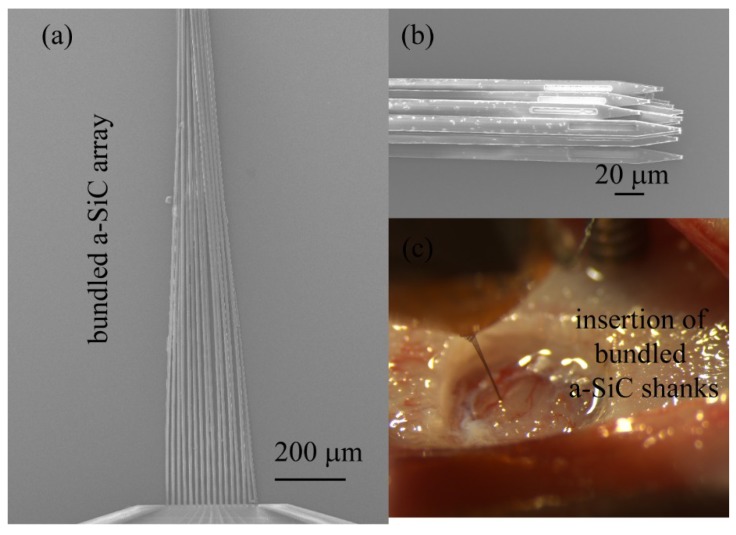
Bundles of 16-channel a-SiC MEA when drawn out of water (**a**) showing tip geometry (**b**). Insertion of a bundled 8-channel a-SiC array into rat cortex (**c**).

Another successful approach introduced by Guitchounts et al. when working with carbon fiber ultramicroelectrodes was to draw the fibers into a bundle allowing the individual fibers to provide mechanical support to each other during array insertion [4]. This approach also increases the overall cross-sectional area of the bundled fibers and increases the buckling threshold for insertion. Since the fibers on the bundled array are held together by weak Van der Waals forces, they separate upon insertion and spread out into the brain following the path of least resistance defined by the mechanical heterogeneity of the brain [4]. The 4 µm thick a-SiC arrays were successfully inserted using this approach, however unlike carbon fibers, we observed that the shanks of the a-SiC MEA twisted together or intertwined when drawn out of water. The tangled shanks prevented the individual shanks from separating and splaying when implanted. Further work is needed to find an appropriate surface treatment that would aid shank separation. Figure 4a shows a bundled a-SiC array formed when the shanks are drawn out of water. Figure 4b shows the tip geometry of the bundle and Figure 4c shows a bundled 8-channel a-SiC array prior to rat cortical implantation.

#### 3.1.3. Reduction of Effective Shank Length

Another factor that influences the critical buckling load is the effective length of the shanks. The effective length of a beam or a shank corresponds to the distance between the points of inflection in the buckled mode. The buckling threshold increases with decreasing effective length of the shank. Patel et al. [5], while working with carbon fibers, developed silicon support structures that enabled the insertion of 0.5 mm long carbon fibers to deeper structures within rat brain. For the carbon fibers, this was the minimum length that could be inserted into the brain without buckling [5]. The advantage of the a-SiC technology over the carbon fiber approach is that structures that will reduce the effective length of the shanks can be designed as part of the MEA geometry. The a-SiC thin film technology allows in situ designs in the a-SiC without the need for additional support structures and micro-assembly. As a result, shorter ultrathin a-SiC array shanks can be developed for insertion into deeper structures within the brain.

**Figure 5:**
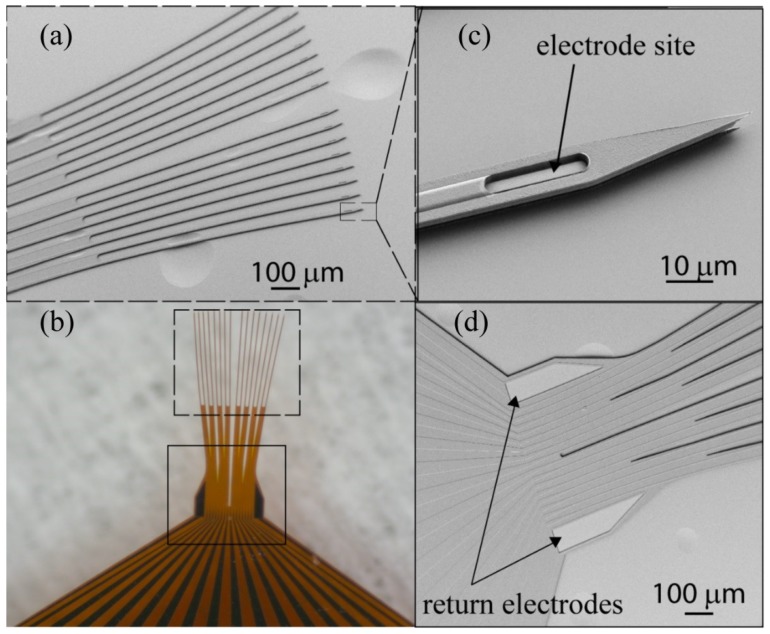
Webbed a-SiC MEA with an effective shank length of 1 mm and a monopolar stimulation current return electrode as part of the MEA structure.

We designed and developed webbed a-SiC arrays as shown in Figure 5b with an effective shank length below 1 mm for an overall insertion depth of 2 mm (including the hinged part). The individual shanks are fused in pairs by a-SiC film interconnects as the shanks approach the base of the MEA 5d while maintaining the ultrathin geometries at the distal end 5a. Electrode sites are located at the distal end 5c. Amorphous SiC MEAs with ultrathin shank geometries (4 µm thick × 10 µm wide) have been successfully implanted when the shanks are webbed. This a-SiC lateral interconnect strategy increases the width of the shank towards the base and may induce lateral stresses in tissue and potentially induce host immune response. We are yet to evaluate the chronic response to these arrays. Since the electrode sites are located on shanks that maintain the critical dimension of 10 µm or less, it is expected that the host immune response, at least around the electrode sites, will be minimized.

#### 3.1.4. Insertion of Individual Shanks

A trade-off between flexibility and stiffness is required when developing compliant microelectrode arrays for cortical application [32]. Insertion of ultrathin flexible microelectrodes into neural tissue usually fails during implantation. The flexural rigidity (a product of the Young’s modulus of the material and moments of inertia of the cross-section) of the shank is related to the critical buckling load by Equation (1) where *P_cr_* is the critical buckling load, *E* is the Young’s modulus, *I* is the moment of inertia of cross-section, *l* is the length, and *K* is the column effective length factor (one fixed end, one pinned end = 0.7). A Young’s modulus of 300 GPa was used for numerical calculations and simulation purposes. The Young’s modulus of a-SiC films depends on deposition conditions and values between 150 and 321 GPa have been reported in the literature [47,48,49].

(1) $$\;P_{cr}=\frac{\pi^{2}EI\;}{{\left(Kl\right)}^{2}}\;$$

The critical buckling load is the maximum axial load a shank can experience that will not cause lateral deflections. For a microelectrode shank to successfully penetrate the pia mater of a rat brain it is generally expected that its critical buckling load to be larger than tissue insertion force estimated to be approximately 0.5 to 2 mN [50,51,52,53,54]. Since the moment of inertia of the cross-section, which influences critical buckling load, depends greatly on the thickness of the shank, COMSOL Multiphysics v. 5.2 (COMSOL AB, Stockholm, Sweden) finite element modeling was used to predict the critical buckling load of a 2 mm long shank when the a-SiC thickness is increased from 4 µm to 6 µm.

**Figure 6:**
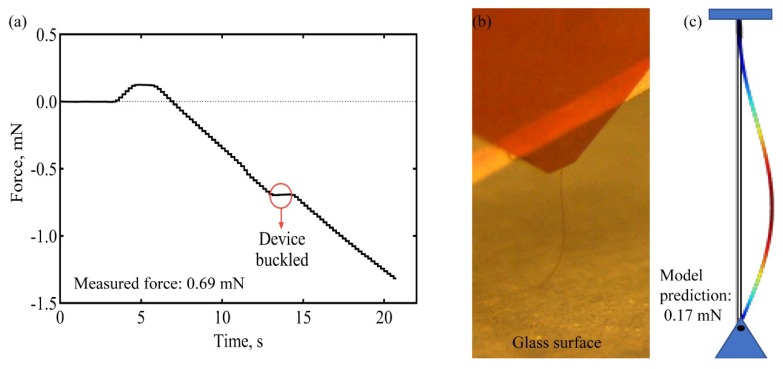
Buckling test. Force measured when a single shank a-SiC probe is lowered against a glass surface (**a**). An image of the buckled state of a 2 mm long shank (**b**) and a COMSOL prediction of the buckled state (**c**).

Force values during a buckling test with a single shank dummy a-SiC probe with a 6 µm thick and 7 µm wide cross-section are shown in Figure 6a. The probe was lowered against a glass surface at a speed of 50 µm/s. No sliding of the probe tip on the glass surface was observed. The lowering was paused when buckling was observed visually, as shown by the plateau at 0.69 mN in Figure 6a. Since the visually observed buckling occurs well-beyond the first deflection of the probe, the 0.69 mN overestimates the buckling force that would be calculated from Equation (1), which is ~0.2 mN for the probe in Figure 6. The recorded buckling force of 0.69 mN should also be adjusted for the nonzero compressive force on the tip when the load cell is tared, which is approximately 0.15 mN. The combined total force of 0.84 mN is notably larger than the COMSOL modeling prediction of 0.17 mN, which is likely due to the uncertainty in the visual assessment of buckling onset and changing boundary conditions as the probe inserts into the brain. The visually observed deflection profile (Figure 6b) was generally in agreement with predictions from the modeling (Figure 6c). Penetration forces are highly dependent on the tip geometry of the implanted device, with larger devices generally exhibiting greater implantation forces. Sridharan et al. [55] measured penetration forces greater than 1 mN using nanocomposite-based devices and observed significant dimpling upon implantation. Welkenhuysen et al. [56] demonstrated penetration forces greater than 0.6 mN using silicon devices, again with significant dimpling.

**Figure 7:**
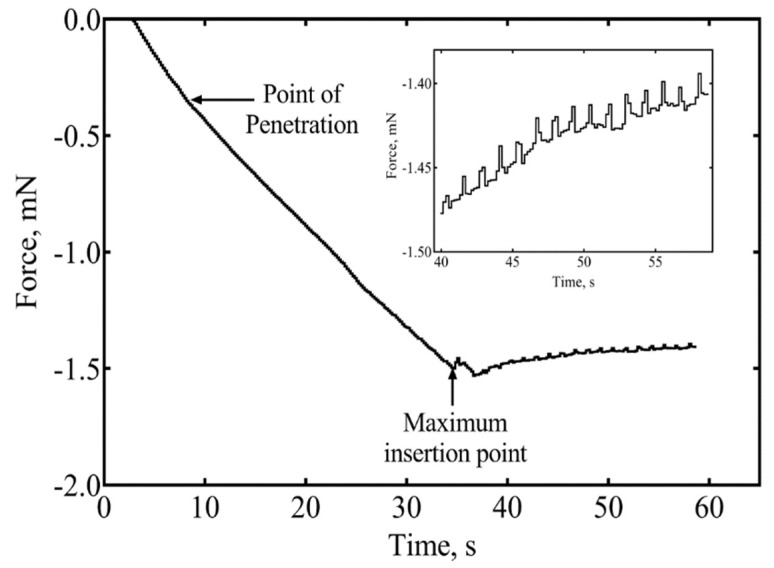
A representative example of the insertion force recorded during the insertion of a 6 µm × 7 µm a-SiC shank into rat cortex. An insertion force of 0.35 mN was recorded at the point of insertion. Inset shows forces experienced by the indwelling shank at 2 mm insertion depth.

A preliminary investigation of the forces involved in inserting a single a-SiC shank into rat cortex was conducted. The force-time curve during implantation of a single shank with a 6 × 7 µm^2^ cross-section at 50 µm/s is shown in Figure 7. From the curve, the point of penetration of the probe corresponds to an insertion force of 0.35 mN. Dimpling of the cortex was not evident. The maximum length of a 6 × 7 μm probe that can be inserted into brain without buckling is 1.4 mm based on Equation (1), using an insertion force of 0.35 mN, an a-SiC modulus of 300 GPa, and *K* = 0.7, corresponding to boundary conditions at which the probe is pinned at the probe-brain interface and fixed at the proximal end. The calculated length likely underestimates that actual length that can be inserted without buckling. As the sharp tips of the probe penetrate the brain, the boundary condition at the probe-brain interface changes to a less challenging fixed condition and the effective length of the shank also decreases slightly. Forces due to brain micromotion (inset) after the probe was implanted to the full 2 mm depth show that the indwelling shank experience an extremely low tissue force which relaxed at a rate of ~2.2 µN/s. We have successfully implanted a single shank and multiple colinear shanks with thickness of 6 µm into a 0.6% agarose gel phantom and into rat brain at an insertion rate of 50 µm/s. To prevent the a-SiC arrays from forming bundles, a minimum intershank distance of 100 µm was found necessary for the 7–10 µm wide shanks investigated. The data in Figure 7 represent the results of a single measurement only and additional studies are required to more fully quantify the forces involved in insertion of these devices into cortex, particularly with respect to the effects of tip geometry, shank cross-sectional dimensions, and shank length. 

### 3.2. Neural Recording

**Figure 8:**
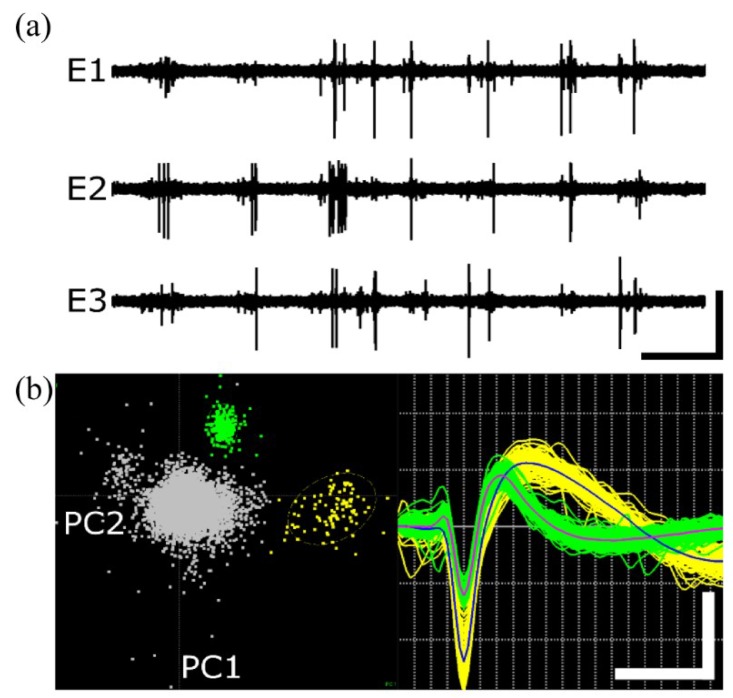
Acute extracellular action potentials recorded using 6 µm a-SiC MEAs. (**a**) Filtered continuous data traces from three representative electrodes on Array 1. Vertical and horizontal scale bar represents 125 µV and 1.75 s, respectively. (**b**) Left—Representative 2D principal component space indicating clear separation from the noise (central gray cluster). Right—Associated single units, indicating characteristic extracellular waveform shape. Vertical and horizontal scale bar represents 175 µV and 0.6 ms, respectively.

To determine whether 6 µm a-SiC and SIROF MEAs could be used for in vivo single-unit extracellular recordings, we performed 10-min electrophysiological recordings immediately following implantation. Figure 8a shows three representative filtered continuous recordings from a single a-SiC array. Extracellular spikes were well-resolved and sorted based on characteristic waveform shape and 2D PC-space clustering into single units (Figure 8b). We observed distinguishable single units on between 25 and 75% of electrode sites, with the total number of units ranging from 4 to 16. These units had mean peak-to-peak amplitudes ranging from 118.5 to 287.7 µV, with a mean amplitude of 179.4 ± 18.4 µV and SNR of 24.1 ± 2.2. Table 1 contains RMS noise, mean amplitude, and SNR values for all three implanted arrays as well as cumulative means. These data suggest that 6 µm a-SiC MEAs are stiff enough to penetrate the cortex without compromising their mechanical/electrical stability and their ability to record single-unit activity.

## 4. Conclusions

The a-SiC platform allows a wide design space to create next generation ultrathin neural interfaces. To reduce overall impedance associated with small electrodes of small geometric surface area, electrode sites could also be coated with common low impedance coating materials, such as TiN or SIROF which decrease the impedance by 2 orders of magnitude over a range of frequencies [35]. For MEAs developed with an overall a-SiC thickness of 4 µm, we have described various techniques which increase the critical buckling force of the individual shanks and enable penetration of the shanks without buckling. These methods include the addition of a temporary stiffening structure, bundling the individual shanks, or through in situ designs which reduced the effective length of the shanks while allowing for targeted depth penetration. With just the addition of a minimal amount of a-SiC material to a thickness of 6 µm, individual single shanks or colinear 2 mm long a-SiC fibers were successfully implanted into rat cortex without buckling. We have also demonstrated the ability to record neural signals using 6 µm thick a-SiC MEAs acutely in rat motor cortex. Our results also indicated that SIROF-coated sites showed high amplitude and high SNR of the recorded neural signals.

## Figures and Tables

**Table 1:** Active electrode yield (AEY) percentage, total number of units, mean peak-to-peak amplitude, RMS noise, and SNR per array, and cumulative values across all arrays.

Array #	AEY (%)	# of Units	Mean Vpp (μV)	RMS Noise (μV)	SNR
Array 1	75	16	179.0 ± 19.8	10.2 ± 1.8	25.6 ± 2.9
Array 2	25	4	287.7 ± 64.4	8.8 ± 0.2	30.8 ± 6.8
Array 3	31.3	7	118.5 ± 12.2	7.8 ± 0.4	16.7 ± 1.7
Cumulative	43.75%	27	179.4 ± 18.4	8.9 ± 0.6	24.1 ± 2.2
